# The *Fbn1* gene variant governs passive ascending aortic mechanics in the mgΔ^*lpn*^ mouse model of Marfan syndrome when superimposed to perlecan haploinsufficiency

**DOI:** 10.3389/fcvm.2024.1319164

**Published:** 2024-03-13

**Authors:** Samar A. Tarraf, Rodrigo Barbosa de Souza, Ashley Herrick, Lygia V. Pereira, Chiara Bellini

**Affiliations:** ^1^ Department of Bioengineering, Northeastern University, Boston, MA, United States; ^2^ Department of Genetics and Evolutionary Biology, University of São Paulo, São Paulo, Brazil

**Keywords:** ascending aneurysm, biomechanics, Marfan syndrome, fibrillin-1, perlecan

## Abstract

**Introduction:**

Ascending thoracic aortic aneurysms arise from pathological tissue remodeling that leads to abnormal wall dilation and increases the risk of fatal dissection/rupture. Large variability in disease manifestations across family members who carry a causative genetic variant for thoracic aortic aneurysms suggests that genetic modifiers may exacerbate clinical outcomes. Decreased perlecan expression in the aorta of mgΔ^*lpn*^ mice with severe Marfan syndrome phenotype advocates for exploring perlecan-encoding *Hspg2* as a candidate modifier gene.

**Methods:**

To determine the effect of concurrent *Hspg2* and *Fbn1* mutations on the progression of thoracic aortopathy, we characterized the microstructure and passive mechanical response of the ascending thoracic aorta in female mice of four genetic backgrounds: wild-type, heterozygous with a mutation in the *Fbn1* gene (mgΔ^*lpn*^), heterozygous with a mutation in the *Hspg2* gene (*Hspg2*^*+/−*^), and double mutants carrying both the *Fbn1* and *Hspg2* variants (dMut).

**Results:**

Elastic fiber fragmentation and medial disarray progress from the internal elastic lamina outward as the ascending thoracic aorta dilates in mgΔ^*lpn*^ and dMut mice. Concurrent increase in total collagen content relative to elastin reduces energy storage capacity and cyclic distensibility of aortic tissues from mice that carry the *Fbn1* variant. Inherent circumferential tissue stiffening strongly correlates with the severity of aortic dilatation in mgΔ^*lpn*^ and dMut mice. Perlecan haploinsufficiency superimposed to the mgΔ^*lpn*^ mutation curbs the viability of dMut mice, increases the occurrence of aortic enlargement, and reduces the axial stretch in aortic tissues.

**Discussion:**

Overall, our findings show that dMut mice are more vulnerable than mgΔ^*lpn*^ mice without an *Hspg2* mutation, yet later endpoints and additional structural and functional readouts are needed to identify causative mechanisms.

## 1 Introduction

Advanced DNA sequencing technology and computational tools have been instrumental for the identification of genetic variants that predispose to aortic aneurysms (1). Insights gained from genetic screening aid the clinical management of patients who suffer from an aortic aneurysm and facilitate early diagnosis in family members, thereby preventing premature deaths (2). A broad range of variants in the *FBN1* gene encoding for fibrillin-1 have been linked to thoracic aortic disease. The glycoprotein fibrillin-1 assembles into microfibrils to guide the formation of elastic fibers and support their long-term stability (3, 4). Defects or mutations in the *FBN1* gene typically give rise to the disarray of elastic fibers in the aorta (4). Since the production of mechanically-competent elastic fibers is limited to the perinatal period, their degradation is often compensated by collagen deposition and reorganization. An increase in the collagen to elastin ratio often leads to tissue stiffening and hinders the aorta from acting as an elastic reservoir to augment diastolic blood flow (5, 6). More than 1,300 mutations in the *FBN1* gene cause Marfan syndrome (MFS), a connective tissue disorder characterized by highly penetrant ascending thoracic aortic aneurysms (ATAAs) (7) that progress to life-threatening aortic rupture and/or dissection in the absence of surgical intervention (8). The gold standard for treatment of ATAAs is open repair surgery, whereby the aneurysmal tissue is excised and replaced with a synthetic vascular graft (9). The current clinical guideline for informing surgical intervention is an aortic diameter exceeding 5cm. However, it has long been observed that diameter is a poor predictor of aortic failure in aneurysmal disease (10, 11). For suitable alternative guidelines to be proposed, a better understanding of disease progression as a function of the genetic underpinnings of ATAAs is necessary.

Evolutionary conservation of the genes linked to thoracic aortic aneurysm warranted the development of mouse models that recapitulate human disease (12) and afford longitudinal studies to monitor tissue microstructure and wall mechanics over time. While several mouse models of MFS exist (13–16), we focus here on the mg$\Delta^{lpn}$ strain (16). mg$\Delta^{lpn}$ mice display classic phenotypic features of patients with MFS, including skeletal kyphosis and ocular defects, as well as enlargement of peripheral air spaces and compromised alveolar wall structures (16–18). Vascular manifestations of MFS in mg$\Delta^{lpn}$ mice encompass thickening of the aortic media, disruption of the elastic fiber network, and formation of thoracic aortic aneurysms. Compared to other mouse models of MFS that tend to die prematurely (13, 19), mg$\Delta^{lpn}$ mice bred on a C57BL/6 background carry a stable phenotype and live up to 9 months, rendering them a suitable model for tracking aortic remodeling and disease progression (16). Toward this end, we first compared biaxial wall mechanics in the ascending thoracic aorta of mg$\Delta^{lpn}$ mice at 4 weeks (pre-aneurysmal), 12 weeks (early aneurysm), and 30 weeks (late-stage aneurysm).

Large intrafamilial variability in the clinical manifestations of MFS (20, 21) suggests that genetic modifiers may exacerbate the phenotype imposed by causative variants. Efforts to identify modifiers genes serve to improve patient screening and recognize individuals at higher risk. It has been shown that mg$\Delta^{lpn}$ mice bred on a 129/Sv background mimic the phenotypic range noted across MFS patients (16). These mice have supported recent attempts at recognizing modifier genes (22, 23) that put forth *Hspg2* as a candidate modifier for MFS (21). *Hspg2* encodes for perlecan, a heparan-sulfate proteoglycan that is present in all basement membranes (24, 25), where it interacts and colocalizes with fibrillin-1 and elastin (26–28). We have recently reported the concurrence of skeletal deformity and ATAA/aortic dissection in mg$\Delta^{lpn}$ mice (17). Furthermore, we noted that mg$\Delta^{lpn}$ mice presenting a severe phenotype with hyperkyphosis, elastic fiber fragmentation, and aneurysm formation, exhibit lower HSPG2 expression in the aorta than mildly affected mice (21). While perlecan-null mice succumb perinatally (29), perlecan deficiency significantly increases the incidence of aortic dissection in a lethality-rescued transgenic model (*Hspg2*${\;}^{-/-}$*–Tg*) (28). Taken together, these findings suggest a link between HSPG2 deficiency and disease severity that warrants further investigation. To explore the role of perlecan haploinsufficiency on early-stage thoracic aortopathy, we characterized biaxial wall mechanics in the ascending thoracic aorta of 12-week-old *Hspg2${\;}^{+/-}$* (29) and age-matched double-mutant mice carrying mutations in both the *Hspg2* and *Fbn1* genes.

## 2 Materials and methods

### 2.1 Animal models

**Figure 1:**
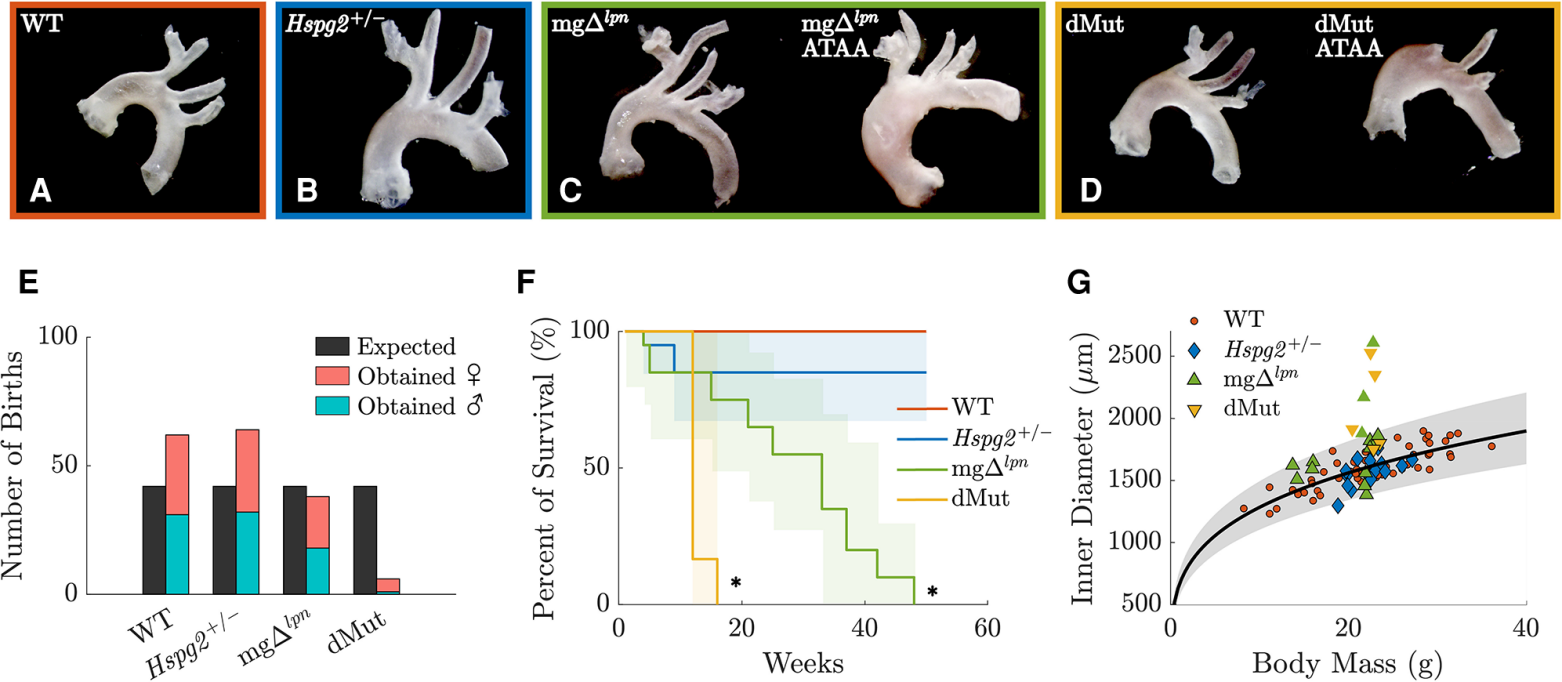
Representative gross anatomy images of the wild-type (WT) control, *Hspg2${\;}^{+/-}$*, mg$\Delta^{lpn}$ (regular-sized and dilated), and double-mutant (dMut; regular-sized and dilated) ascending thoracic aorta (ATA) following excision from 12-week-old mice (**A**–**D**). The yield of dMut mice in the offspring of heterozygous *Fbn1*/mg$\Delta^{lpn}$ and *Hspg2${\;}^{+/-}$* mating pairs falls below expected Mendelian ratio and includes mostly (5/6) female mice (**E**). Kaplan–Meier curves show normal lifespan in *Hspg2${\;}^{+/-}$* mice and reduced survival in mg$\Delta^{lpn}$ mice. Confidence intervals and statistical significance for the Kaplan–Meier curves assessed with the Greenwood’s formula, with * for p<0.05 (**F**). Allometric scaling relating body mass to luminal diameter for the WT control ATA (black line) supports definition of an expected range of aortic sizes within a 95% confidence interval (gray shaded area). Overall, n=3 mg$\Delta^{lpn}$ and n=3 dMut ATA samples at the 12-week endpoint fall outside normal allometric scaling and are considered dilated. However, only 2 of the dilated ATA samples exceed the current clinical threshold for aneurysmal diagnosis that is set to normalized diameter above 1.5 (**G**).

All animal experiments followed protocols approved by the Institutional Animal Care and Use Committee of the Instituto de Biociências at the University of São Paulo (USP). Mice were bred from heterozygous mg$\Delta^{lpn}$ (*Fbn1*/mg$\Delta^{lpn}$) and *Hspg2${\;}^{+/-}$* mating pairs on a C57BL/6 background, with genotyping performed as described in (16). Mice were kept under controlled conditions of light and temperature in a pathogen-free environment at the Central Animal Facility of the School of Medicine at USP. Female, 12-week-old offspring were organized by genotype in four experimental groups: wild-type (WT, n=14; Figure 1A), perlecan haploinsufficient (*Hspg2${\;}^{+/-}$*, n=9; Figure 1B), Marfan syndrome (mg$\Delta^{lpn}$, n=9; Figure 1C), and double-mutant (dMut, n=5; Figure 1D) mice. In addition, 4-week-old (n=4) and 30-week-old (n=2) female mg$\Delta^{lpn}$ mice were used for monitoring aneurysmal disease over time and compared to age-matched controls (4-week-old WT, n=4; 30-week-old WT, n=3). Note, we focused the study on female mice as dMut mice of male sex were not viable, with only a single male dMut mouse born out of 170 offspring over the course of a year (Figure 1E). Moreover, the slower disease progression in female vs. male mg$\Delta^{lpn}$ mice permitted tracking progressive aortic dilatation between 4 and 30 weeks-of-age. The 12-week endpoint specifically granted inclusion of mice presenting with different degrees of aortic dilatation. After sacrifice, the thoracic aorta of each mouse was excised from the thoracic cavity, stored in Phosphate-Buffered Saline (PBS) solution, and shipped over dry ice to Northeastern University for mechanical testing. After arriving at Northeastern, samples were kept in a −80${\;}^{\circ }$C freezer. Prior to testing, samples were thawed overnight in a 4${\;}^{\circ }$C fridge. Preliminary testing indicated no significant differences in passive mechanics between frozen aortic samples and fresh tissue (30, 31).

### 2.2 Passive mechanical testing

All biomechanical testing and data analysis was performed consistent with established methods (12, 32, 33, 34). The thoracic aorta was cleaned of excess fat and perivascular tissues, while preserving lateral branches. Branches were ligated using 9-0 nylon sutures. The ascending thoracic aorta (ATA) was isolated from the aortic root to the left subclavian artery. ATA segments were cannulated at each of the aortic root and brachiocephalic artery ends onto custom-pulled glass micropipettes and secured using double knots of 6-0 silk sutures. Cannulated specimens were then attached to a custom-built, computer-controlled biaxial mechanical testing device (35) and submerged in Hank’s Balanced Salt Solution (HBSS). Luminal pressure was regulated via a hydraulic circuit connected to both cannulae. The outer diameter of each sample was recorded throughout testing using a camera positioned in front of a transparent bath in which the vessel was submerged. Linear actuators adjusted the distance between the two cannulae, thereby modifying the axial stretch, while a load cell coupled to the distal cannula measured the axial force. Luminal pressure and axial length were controlled through a custom LabView (National Instruments, Austin, TX) interface.

The *in-vivo* axial length of each sample was estimated by gradually stretching the tissue while varying the pressure, to find the energetically optimal stretch at which the axial force remains constant with pressure (36). Vessels were first acclimated via a pulsatile luminal pressurization from 80 to 120 mm Hg while held at their *in-vivo* axial length, then preconditioned using 4 pressurization cycles between 10 and 140 mm Hg. After re-establishing the traction-free geometry and the *in-vivo* axial stretch, vessels were subjected to pressure-diameter and force-length tests. In the former protocol set, vessels were held at the estimated *in-vivo* stretch or ±5% of this value, while luminal pressure varied cyclically between 10 and 140 mm Hg. In the latter protocol set, luminal pressure was maintained constant at 10, 60, 100, or 140 mm Hg, while specimens were stretched to vary the axial force between −0.5 and 5 g. Each test is comprised of two cycles, with data collected on the second cycle. Upon completion of all protocols, samples were removed from the biaxial device and a thin ring was cut just distal of the aortic root and imaged with a dissection microscope for estimation of traction-free wall thickness using a custom MATLAB script (MATLAB R2022b; Mathworks, Natick, MA).

### 2.3 Mechanical analysis

Passive biaxial data were fit via nonlinear regression to the following 8-parameter stored energy function (12, 32)

(1) $$W(C,M^{i})=\frac{c}{2}(I_{C}-3)+\sum_{i=1}^{4}\frac{c_{1}^{i}}{4c_{2}^{i}}\{\exp[c_{2}^{i}(IV_{C}^{i}-1{)}^{2}]-1\},$$

where c and $c_{1}^{i}$ are material parameters with the units of stress, while $c_{2}^{i}$ are dimensionless parameters; $C=F^{T}F$ is the right Cauchy-Green tensor calculated from the deformation gradient F; $M^{i}=[0,\sin\alpha_{0}^{i},\cos\alpha_{0}^{i}]$ is a unit vector in the direction of the $i^{th}$ fiber family, with the angle $\alpha_{0}^{i}$ defined relative to the axial direction in the reference configuration (i=1 axial, i=2 circumferential, and i=3,4 symmetric diagonal); $I_{C}=\mathrm{tr}(C)$ and $IV_{C}^{i}=M^{i}CM^{i}$ are invariant measures of the deformation. In cylindrical coordinates, the deformation gradient F admits matrix form $\mathrm{diag}[\lambda_{r},\lambda_{\theta },\lambda_{z}]$, with $\lambda_{r}$ determined by enforcing incompressibility (J=1) such that $\lambda_{r}=1/\lambda_{\theta }\lambda_{z}$.

Given best fit parameters for the strain energy density function (Eq. 1), the Cauchy stress tensor for any given deformation is

(2) $$\sigma =-pI+2F\frac{\partial W(C)}{\partial C}F^{T},$$

where p is a Lagrange multiplier and I is the identity tensor.

Components of the linearized stiffness tensor were determined according to the small-on-large deformation approach (37)

(3) $$C_{ijkl}=\sigma_{il}\delta_{\;jk}+\sigma_{lj}\delta_{ik}+4F_{iI}F_{\;jJ}F_{kK}F_{lL}\frac{\partial^{2}\hat{W}(C)}{\partial C_{IJ}\partial C_{KL}},$$

where δ is the Kronecker delta, and the indices (i,j,k,l)≡(r,θ,z) and (I,J,K,L)≡(R,Θ,Z) refer to the current and reference configurations, respectively.

Transmural pressures of 80mmHg and 120mmHg were considered representative of *in-vivo* diastolic and systolic loads, respectively. Geometry, energy storage, biaxial stretch and stress, and tissue stiffness were computed at systolic pressure and individual values of the estimated *in-vivo* stretch. Cyclic aortic distensibility, an inverse measure of structural stiffness, was calculated as

(4) $$D=\frac{d_{i,sys}-d_{i,dia}}{d_{i,dia}(P_{sys}-P_{dia})}$$

with P and $d_{i}$ denoting luminal pressure and diameter, respectively, and the subscripts sys and dia indicating systole and diastole.

### 2.4 Allometric scaling

Luminal aortic diameter ($d_{i}$) scales allometrically with body mass (BM), according to the relationship (38)

(5) $$d_{i}=\alpha \cdot BM^{\beta }.$$

The coefficients α and β were determined by linear regression on predicted luminal diameters at systole for female C57BL/6 WT mice in this study pooled with prior data by our group (33). Vessels that fell beyond the 95% confidence interval for normal allometric scaling were classified as dilated. Mechanical metrics were compared against aortic dilatation, with normalized diameter defined as the ratio between predicted luminal diameter and expected diameter based on body mass from allometric scaling.

### 2.5 Histology and immunofluorescence

Following passive mechanical testing, aortic samples were embedded in optimal cutting temperature (OCT) compound (4585, Fisher Scientific), fast-frozen, and stored at −80${\;}^{\circ }$C. Tissues were cryosectioned to 6 μµm in thickness and placed on SuperFrost Plus slides (Thermo Fisher Scientific, Inc., Waltham, MA) with three cross sections per aortic sample. Slides were stained with Verhoeff–Van Gieson (VVG) or picrosirius red (PSR) stains and imaged under bright field or polarized light, respectively, using a 40× objective (DM4 B upright microscope, Leica microsystems). Following stitching (Image Composite Editor, Microsoft 2015), aortic rings were analyzed using a custom MATLAB script (MATLAB R2022b; Mathworks, Natick, MA). Remaining slides were fixed for 10 minutes with 10% neutral buffer, rinsed with 1X PBS, then blocked and permeablized with 5% BSA and 0.1% Tween in 1X PBS for 30 min. Slides were rinsed and then incubated for two hours with the following primary antibodies: anti-collagen I (1:250, Abcam, ab270993), anti-collagen III (1:200, Abcam, ab7778), and anti-collagen IV (1:100, Abcam, ab235296). Following rinsing with PBS, slides were incubated with the appropriate secondary antibody for 1 h. Secondary antibodies consisted of donkey anti-rabbit Alexa Flour 647 (1:500, Abcam, ab150075) and donkey anti-goat Alexa Flour 568 (1:500, Abcam, ab175704). Slides were further stained with DAPI (1:5000, Thermo Scientific, 62,248) for 10 min to allow for visualization of cell nuclei. Two regions per cross section were imaged at 20× using a Zeiss LSM 800 confocal microscope and Zeiss Efficient Navigation (ZEN) software (Carl Zeiss Microscopy, Jena, Germany), using the same acquisition parameters per stain across groups and leveraging the autofluorescence of elastin.

### 2.6 Statistics

All values are reported as mean ± standard error of mean (SEM). Since mechanical and histological data were normally distributed as assessed by the Shapiro–Wilk test, differences across groups were determined via one-way ANOVA with post-hoc Tukey test for multiple comparisons. We further relied on the non-parametric Spearman’s rank-order correlation test to probe the relationship between mechanical metrics and aortic dilation, where $r_{s}$ describes the strength and direction of the correlation. Correlations were deemed mild, moderate, and strong for $|r_{s}|\leq 0.5$, $0.5<|r_{s}|\leq 0.75$, and $|r_{s}|>0.75$, respectively.

## 3 Results

*Allometric scaling facilitated segregation of dilated from regular-sized ATAs.* Allometric scaling was used to normalize luminal ascending thoracic aortic (ATA) diameter by body mass. A logarithmic transformation followed by linear regression of predicted systolic diameter for n=56 ATA samples from female C57BL/6 wild-type mice yielded best-fit values of $\alpha =671.7862\;m\cdot g^{\beta }$ and β=0.2816 (Eq. 5). These estimates are in close agreement with experimental (33, 39, 40) and theoretical (38) values from literature. All 12-week-old *Hspg2${\;}^{+/-}$* specimens fell within the 95% confidence interval for the expected ATA diameter by body mass (Figure 1G). A subset of mice carrying the *Fbn1* variant featured ATA dilatation (Figure 1G), including approximately one third (3 out of 9) of the mg$\Delta^{lpn}$ and half (3 out of 5) of the dMut mice at the 12-week endpoint. None of the 4-week-old and all of the 30-week-old ATA samples from mg$\Delta^{lpn}$ mice were dilated. Note that only 4 out of 8 dilated ATA samples (including vessels from both the 12- and 30-week endpoints) exceeded the current clinical threshold for aneurysmal diagnosis, i.e., they exhibited normalized diameter above 1.5 (41).

**Figure 2:**
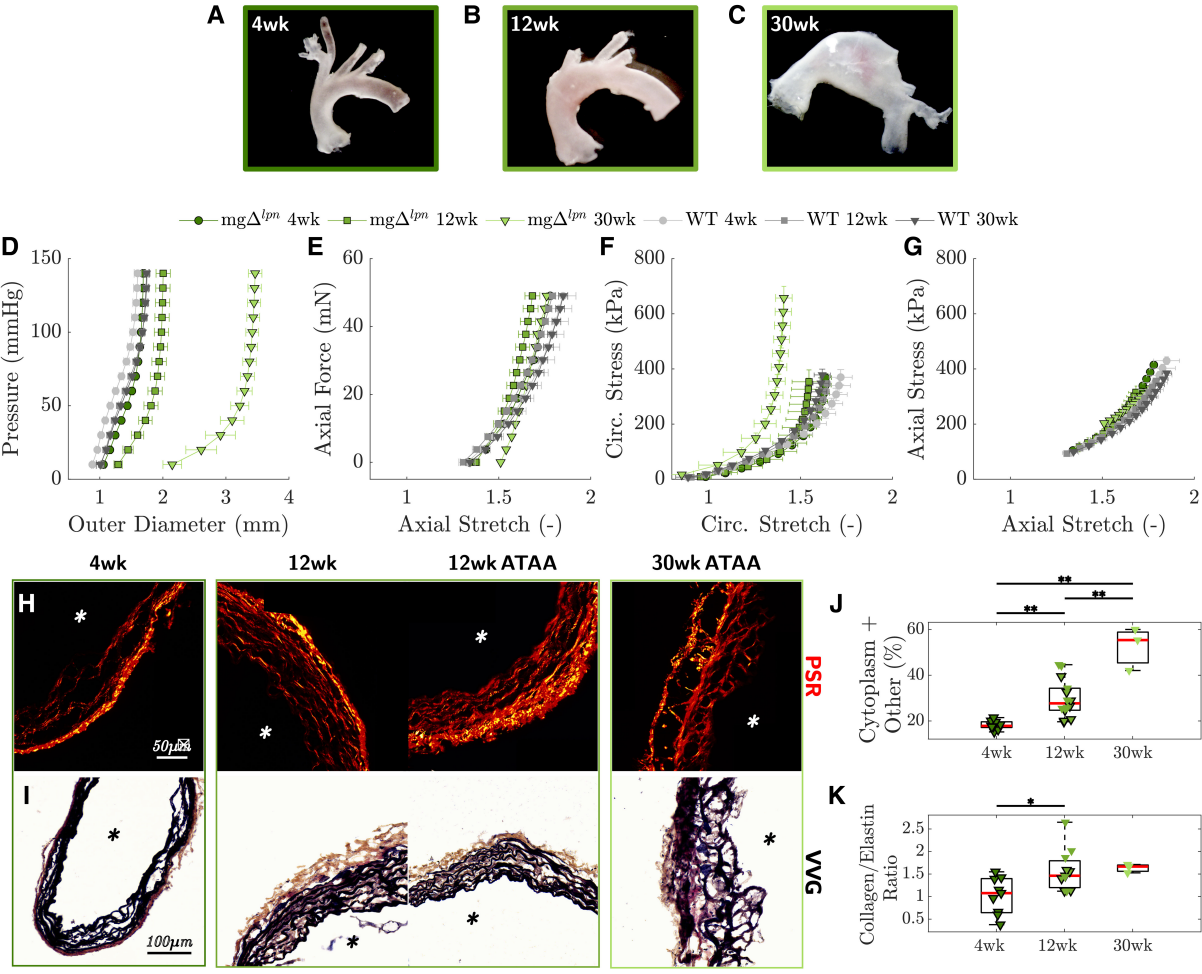
Representative gross anatomy images of the ascending thoracic aorta (ATA) following excision from mg$\Delta^{lpn}$ mice show progression of aortic dilatation from 4 to 12 weeks-of-age and development of advanced aneurysm by 30 weeks (**A**-**C**). Average pressure vs. diameter (**D**) and axial force vs. length (**E**) responses reveal gradual decline of circumferential distensibility and axial extensibility that reflect the leftward shift of biaxial stress vs. stretch responses in the circumferential (**F**) and axial (**G**) directions, compared to age-matched wild-type (WT) controls. Anticipation/loss of the inflection point in the pressure vs. diameter behavior is already apparent by the 4-week endpoint and therefore precedes dilatation. Picrosirius red (PSR, **H**) and Verhoeff–Van Gieson (VVG, **I**) stains visualize the architecture of collagen and elastic fibers in ATA tissues from mg$\Delta^{lpn}$ mice. The progressively higher proportion of cytoplasm and tissues other than elastin in VVG-stained cross sections follows the loss of elastic fibers that occurs with aneurysmal enlargement from 4 to 30 weeks-of-age (**J**). The ratio of collagen to elastin content likewise increases as mg$\Delta^{lpn}$ mice age (**K**). Asterisks visualize the intimal side in histological cross sections. Markers without a black outline indicate dilated vessels in the boxplot. Statistical significance denoted by overbar, with * for p<0.05 and ** for p<0.01.

*Structural stiffening of the ATA progressed with aneurysmal dilatation in mg$\Delta^{lpn}$ mice.* Despite not yet presenting with an aneurysm (Figure 2A), the ATA of 4-week-old mg$\Delta^{lpn}$ mice was structurally stiffer compared to age-matched wild-type (WT) control. The mg$\Delta^{lpn}$ mutation abrogated the inflection point that marks the change in convexity in the circumferential pressure vs. diameter behavior of the ATA (Figure 2D), while concurrently reducing axial extensibility in the force vs. stretch response (Figure 2E). Aligned with structural observations, both the circumferential (Figure 2F) and axial (Figure 2G) stress vs. stretch behavior of tissues from the 4-week-old mg$\Delta^{lpn}$ ATA remained confined to the left of WT control, delivering comparable stress at lower deformation. Moderate dilatation of the mg$\Delta^{lpn}$ ATA at 12 weeks (Figure 2B) coincided with further loss of circumferential distensibility (Figure 2D) and axial extensibility (Figure 2E). By 30 weeks-of-age the mg$\Delta^{lpn}$ ATA became aneurysmal (Figure 2C) and featured inherently stiffer tissues that developed higher circumferential stress compared to WT control when extended to the same stretch (Figure 2F). Best fit material parameters at all three endpoints are listed in Table 1, while descriptors of structural and material properties are reported in Supplementary Material Table S1. Consistent with structural and circumferential tissue stiffening, the ratio of collagen (Figure 2H) to elastin (Figure 2I) content within ATA tissues increased as mg$\Delta^{lpn}$ mice aged from 4 to 12 weeks, and remained elevated up to the 30-week endpoint (Figure 2K). Meanwhile, the relative content of non-elastin tissue elements, including cell cytoplasm, in VVG-stained tissue cross sections also reached higher values (Figure 2J).

**Table 1:** Best-fit material parameters for the microstructurally motivated strain energy function in Eq. 1 that describes the representative mechanical response of ascending thoracic aortic (ATA) tissues from wild-type (WT) control, *Hspg2${\;}^{+/-}$*, mg$\Delta^{lpn}$, and double-mutant (dMut) mice.

	Age (wks)	Elastic fibers c (kPa)	Ax. collagen		Circ. collagen + SMC		Symmetric diag. collagen			Error RMSE
			$c_{1}^{1}$ (kPa)	${c_{2}}^{1}$	${c_{1}}^{2}$ (kPa)	${c_{2}}^{2}$	${c_{1}}^{3,4}$ (kPa)	$c_{2}^{3,4}$	$\alpha_{0}$ (deg)	
WT	4	25.05	15.85	0.04	11.36	$2.34\times 10^{-14}$	11.06	0.40	51	0.108
WT	12	14.46	12.98	0.07	0.03	1.75	29.45	0.20	53	0.064
WT	30	21.48	7.52	$2.22\times 10^{-14}$	9.42	0.45	17.52	0.27	46	0.066
*Hspg2${\;}^{+/-}$*	12	14.77	12.69	0.05	0.01	2.09	32.91	0.18	53	0.065
mg$\Delta^{lpn}$	4	30.07	2.79	0.41	$1.75\times 10^{-10}$	8.57	13.22	0.63	53	0.104
mg$\Delta^{lpn}$	12	$2.32\times 10^{-14}$	14.79	$2.32\times 10^{-14}$	3.31	1.05	23.44	0.44	50	0.150
mg$\Delta^{lpn}$	30	27.78	$2.83\times 10^{-4}$	$6.67\times 10^{-6}$	$1.33\times 10^{-7}$	18.59	21.31	1.58	59	0.111
dMut	12	$5.28\times 10^{-11}$	12.88	$3.24\times 10^{-12}$	2.41	0.63	30.15	0.35	49	0.144

**Figure 3:**
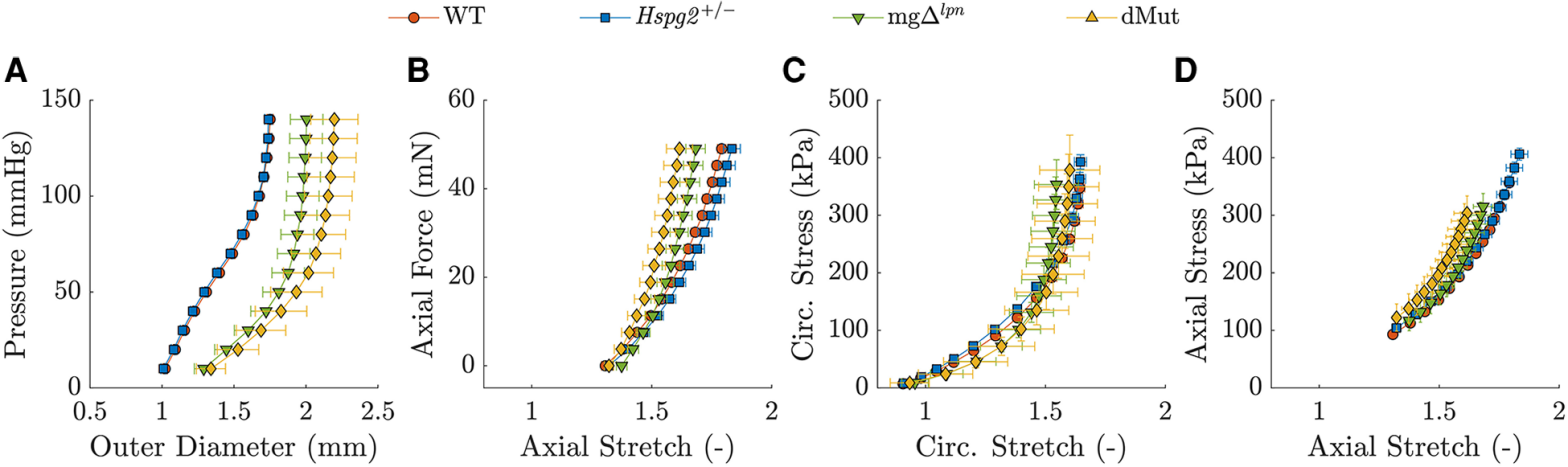
Average pressure vs. diameter (**A**) and axial force vs. length (**B**) responses with associated biaxial tissue behavior in the circumferential (**C**) and axial (**D**) directions for the wild-type (WT) control, *Hspg2${\;}^{+/-}$*, mg$\Delta^{lpn}$ (regular-sized and dilated), and double-mutant (dMut; regular-sized and dilated) ascending thoracic aorta (ATA) following excision from 12-week-old mice. The ATA retains normal global mechanical behavior despite perlecan haploinsufficiency and the mg$\Delta^{lpn}$ mutation remains a stronger determinant of global mechanical behavior compared to perlecan haploinsufficiency in the dMut ATA by 12 weeks-of-age.

*The mg$\Delta^{lpn}$ variant dominated the passive mechanical behavior of the dMut ATA.* The passive structural (Figures 3A,B) and tissue (Figures 3C,D) responses of the perlecan haploinsufficient (*Hspg2${\;}^{+/-}$*) ATA at the 12-week endpoint nearly overlapped with those of age-matched WT controls. Likewise, the global structural (Figures 3A,B) and tissue (Figures 3C,D) behavior of the ATA from mg$\Delta^{lpn}$ and dMut mice was comparable at the 12-week endpoint, although the latter extended to larger diameters. Best fit material parameters for all genotypic groups at 12 weeks-of-age are listed in Table 1.

**Figure 4:**
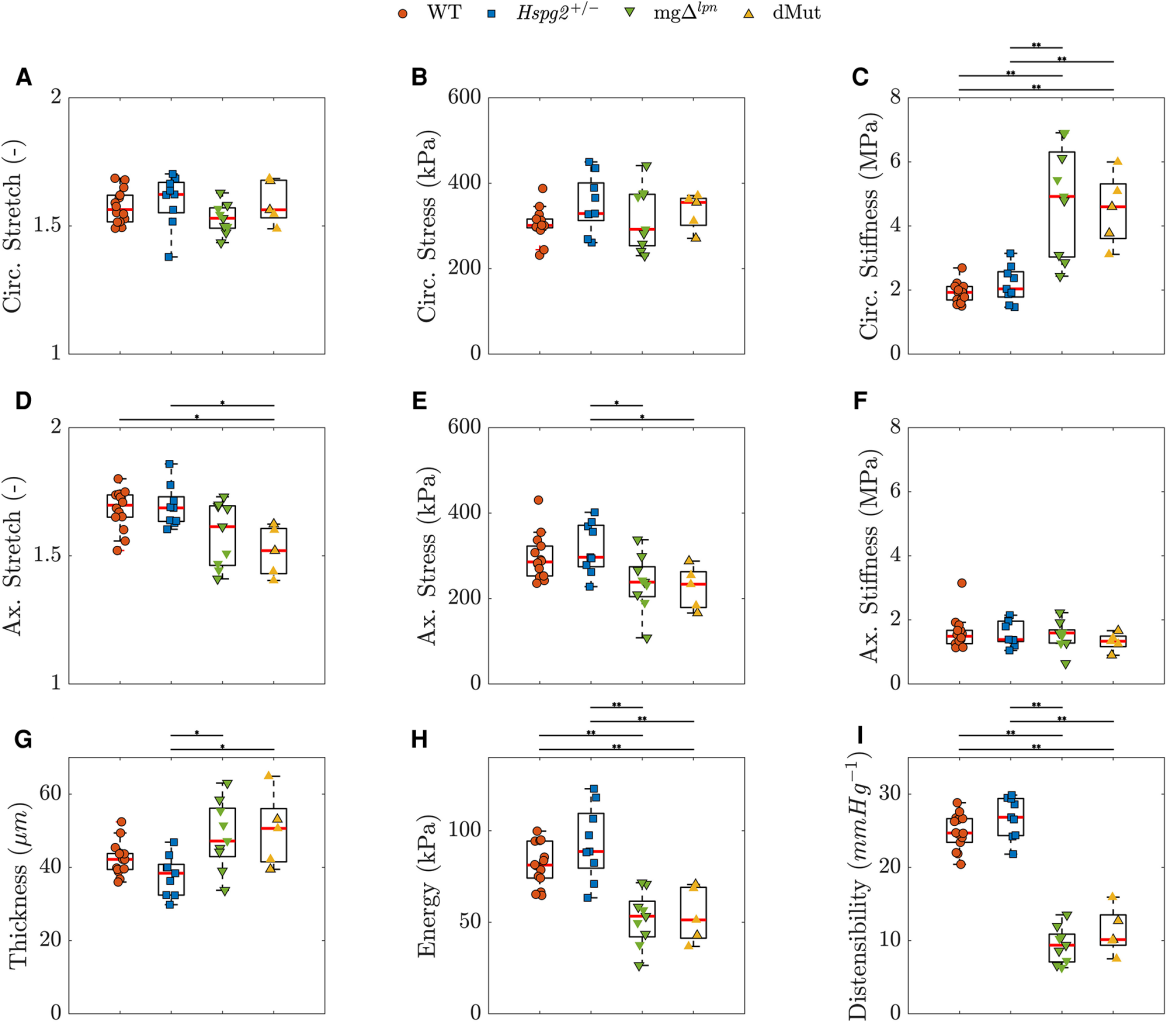
Predicted *in vivo* material, geometrical, and structural properties of the ascending thoracic aorta (ATA) from 12-week-old wild-type (WT) control, *Hspg2${\;}^{+/-}$*, mg$\Delta^{lpn}$ (regular-sized and dilated), and double-mutant (dMut; regular-sized and dilated) mice grouped by genotype. Perlecan haploinsufficiency preserves inferred mechanical metrics in the *Hspg2${\;}^{+/-}$* ATA. The *Fbn1* variant overall supports tissue circumferential stiffening (**C**) and radial thickening (**G**), lowers the axial stress (**E**), limits the capacity for energy storage (**H**), and reduces the cyclic distensiblity (**I**) of the ATA from both mg$\Delta^{lpn}$ and dMut mice. However, the dMut ATA alone exhibits reduced axial stretch (**D**). Circumferential stretch (**A**), circumferential stress (**B**), and axial stiffness (**F**) are remarkably consistent across genotypic groups. Markers without a black outline indicate dilated vessels in the boxplots. Statistical significance denoted by overbar, with * for p<0.05 and ** for p<0.01.

When evaluated at *in-vivo* loads, ATA tissues from WT and *Hspg2${\;}^{+/-}$* mice experienced comparable biaxial stretch, stress (Eq. 2), and stiffness (Eq. 3), demonstrated similar capacity for energy storage, and exhibited matching wall thickness and cyclic distensibility (Eq. 4), (Figures 4A–I). The *Fbn1* variant, either alone or superimposed to perlecan haploinsufficiency, did not affect circumferential stretch levels in ATA tissues (Figure 4A). In contrast, ATA samples from dMut (1.52±0.04) mice alone extended to lower axial stretch compared to their WT (1.68±0.02) and *Hspg2${\;}^{+/-}$* (1.69±0.03) counterparts (Figure 4D; p<0.05). The ATA wall was thicker in mg$\Delta^{lpn}$ (49±3 μµm) and dMut (50±4 μµm) than in *Hspg2${\;}^{+/-}$* (38±2 μµm) mice (Figure 4G; p<0.05). Nevertheless, circumferential stress was remarkably preserved across all groups (Figure 4B), while axial stress was lower in mg$\Delta^{lpn}$ (236±22 kPa) and dMut (225±22 kPa) compared to *Hspg2${\;}^{+/-}$* (318±20 kPa) mice. Axial stiffness did not differ significantly across groups (Figure 4F), while ATA tissues from mice carrying the *Fbn1* variant exhibited higher circumferential stiffness (mg$\Delta^{lpn}$: 4.83±0.57 MPa, dMut: 4.51±0.50 MPa) compared to both WT (1.87±0.09 MPa) and *Hspg2${\;}^{+/-}$* (2.18±0.19 MPa) tissues (Figure 4C; p<0.01). Importantly, reduced capacity for energy storage (WT: 81±3 kPa, *Hspg2${\;}^{+/-}$*: 93±7 kPa, mg$\Delta^{lpn}$: 52±5 kPa, dMut: 54±7 kPa) and cyclic distensibility (WT: 25±1 mm Hg${\;}^{-1}$, *Hspg2${\;}^{+/-}$*: 27±1 mm Hg${\;}^{-1}$, mg$\Delta^{lpn}$: 9±1 mm Hg${\;}^{-1}$, dMut: 11±1) evidenced functional loss in the aorta of mice carrying the *Fbn1* variant (Figures 4H,I; p<0.01). Descriptors of structural and material properties for all genotypic groups at 12 weeks-of-age are listed in Supplementary Material Table S1.

**Figure 5:**
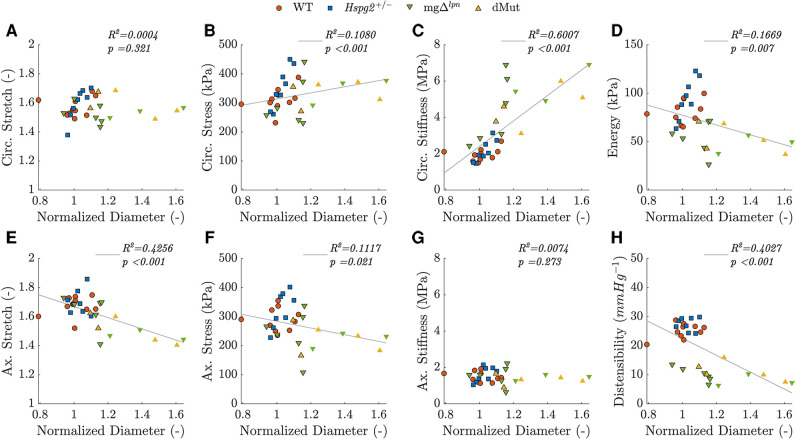
Predicted *in vivo* material, geometrical, and structural properties of the ascending thoracic aorta (ATA) from 12-week-old (WT) control, *Hspg2${\;}^{+/-}$*, mg$\Delta^{lpn}$ (regular-sized and dilated), and double-mutant (dMut; regular-sized and dilated) mice visualized as a function of normalized ATA diameter, i.e., the ratio between current diameter and the expected diameter for a WT mouse with the same body mass. Aortic diameter is strongly and positively correlated with circumferential tissue stiffness ($r_{s}=0.842$, p<0.001; **C**), moderately and negatively correlated with distensibility ($r_{s}=-0.622$, p<0.001; **H**) and axial stretch ($r_{s}=-0.561$, p<0.001; **E**), moderately and positively correlated with circumferential stress ($r_{s}=0.532$, p<0.001; **B**), and mildly and negatively correlated with energy storage capacity ($r_{s}=-0.383$, p<0.01; **D**) and axial stress ($r_{s}=-0.328$, p<0.05; **F**). Aortic diameter is not significantly correlated with either circumferential stretch (**A**) or axial stiffness (**G**). Markers without a black outline indicate a dilated vessel. Note, although Spearman’s rank is a non-parametric measure of correlation that does not assume linearity, best fit lines are added to aid trend visualization for each mechanical metric.

*Intrinsic circumferential tissue stiffness positively correlated with aortic dilatation.* Consistent with previous findings (12), circumferential tissue stiffening strongly correlated with aortic dilatation ($r_{s}=0.842$, p<0.001; Figure 5C). Widening of the lumen moderately correlated with the rise in circumferential stress ($r_{s}=0.532$, p<0.001; Figure 5B) as well as the decline in axial stretch ($r_{s}=-0.561$, p<0.001; Figure 5E). A mild negative correlation further emerged between aortic dilatation and axial stress ($r_{s}=-0.328$, p<0.05, p<0.001; Figure 5F). Functional metrics of cyclic distensibility ($r_{s}=-0.622$, p<0.001; Figure 5H) and energy storage ($r_{s}=-0.383$, p<0.01; Figure 5D) exhibited negative correlations with aortic dilatation. No meaningful correlation was noted between circumferential stretch (Figure 5A) or axial stiffness (Figure 5G) and aortic diameter.

**Figure 6:**
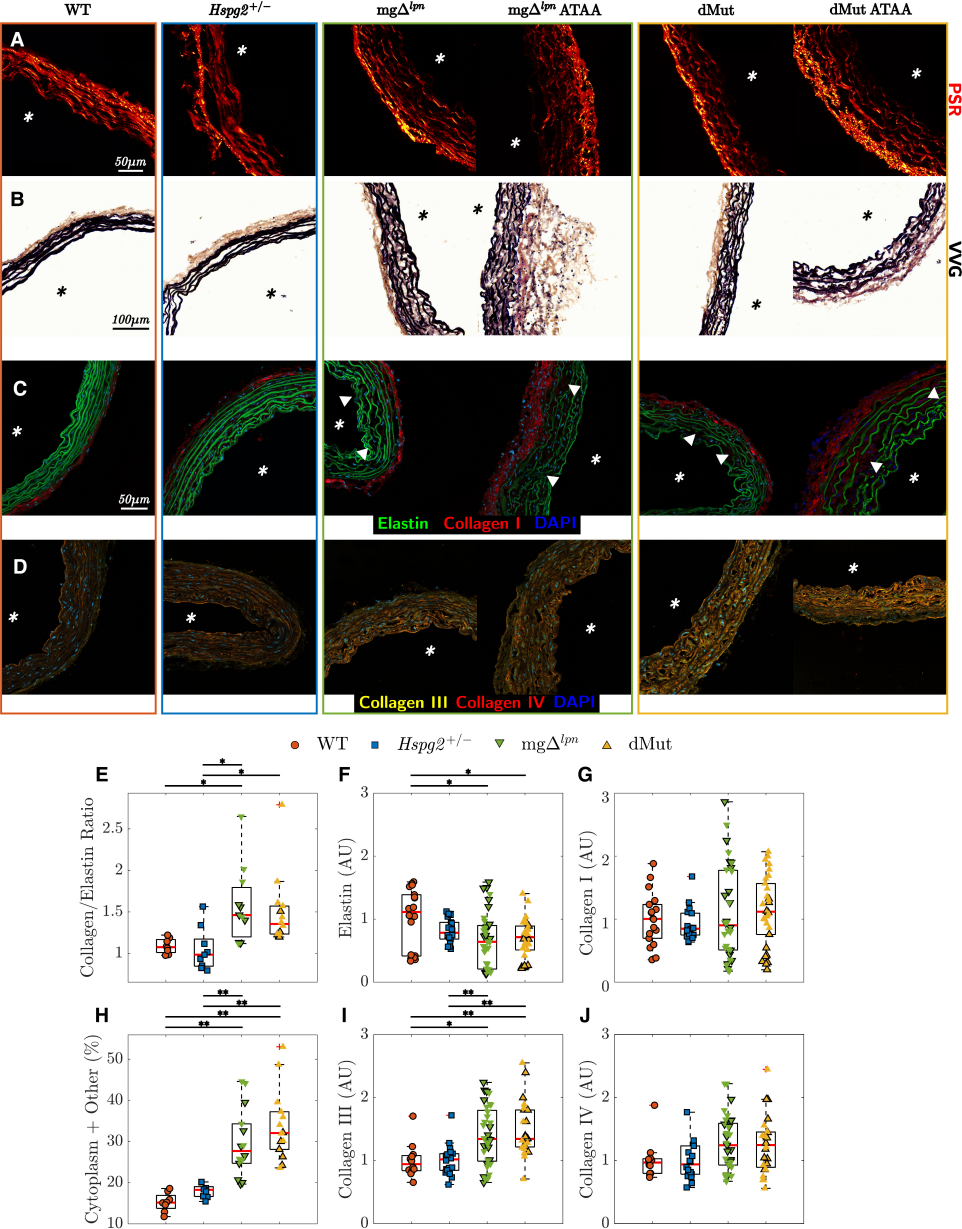
Elastic and collagen fiber architecture in tissues from the ascending thoracic aortic (ATA) of 12-week-old wild-type (WT) control, *Hspg2${\;}^{+/-}$*, mg$\Delta^{lpn}$ (regular-sized and dilated), and double-mutant (dMut; regular-sized and dilated) mice visualized by picrosirius red (PSR, **A**), Verhoeff–Van Gieson (VVG, **B**), and immunofluorescence staining (**C**,**D**). Perlecan haploinsufficiency alone maintains normal organization of elastic and collagen fibers in *Hspg2${\;}^{+/-}$* ATA tissues. Both mg$\Delta^{lpn}$ and dMut mice present ATA cross sections with larger collagen to elastin content ratio (**E**) and higher relative content of cytoplasm and other non-elastin tissues (**H**), compared to WT and *Hspg2${\;}^{+/-}$* mice. Consistently, the mean fluorescence intensity of elastin by area (**F**) decreases, yet the mean fluorescence intensity of collagen III by area (**I**) increases in the ATA of mice carrying the *Fbn1* variant. The mean fluorescence intensity of collagen I (**G**) and collagen IV (**J**) by area is comparable across all genotypes. Asterisks visualize the intimal side in histological cross sections, and white triangles show fragmentation, gaps, or disarray in the elastic fiber network. Markers without a black outline indicate dilated vessels in all boxplots. Statistical significance denoted by overbar, with * for p<0.05 and ** for p<0.01.

*Increased tissue accumulation of collagen III compensated for elastic fiber degradation in the dMut ATA.* Supporting mechanical findings, ATA tissues collected from WT and *Hspg2${\;}^{+/-}$* mice exhibited similar microstructure at the 12-week endpoint (Figures 6A–D), with preserved collagen to elastin ratio (Figure 6E), competent elastic fiber network (Figure 6F), and comparable collagen I, III, and IV accumulation (Figures 6G,I,J). ATA tissues from 12-week-old mg$\Delta^{lpn}$ and dMut mice followed consistent patterns of microstructural disarray that worsened with the severity of aortic dilatation (Figures 6A–D). The ratio of collagen to elastin content was higher in mg$\Delta^{lpn}$ and dMut mice compared to WT control and *Hspg2${\;}^{+/-}$* mice, respectively (Figure 6E). Similarly, the relative content of cytoplasm and other tissues in VVG-stained cross sections was larger in mice carrying the *Fbn1* variant (Figure 6H) contrasted to both WT control and *Hspg2${\;}^{+/-}$* mice. Qualitative interrogation of the ATA wall microstructure via immunofluorescence staining revealed gradual loss of elastin integrity that began at the inner elastic lamina (IEL) and expanded outward in mg$\Delta^{lpn}$ and dMut mice. Disarray of elastic fibers in the proximity of the IEL became apparent in regular-sized ATA tissues from 12-week-old mice, while fiber fragmentation extended toward the adventitial interface in mice of the same age presenting with ATA dilatation (Figure 6C). This coincided with a decrease in mean elastin fluorescence intensity in both genotypes carrying the *Fbn1* variant compared to WT controls (Figure 6F). Unlike collagen I and IV (Figures 6G,J), collagen III accumulation was enhanced in the aortic media of ATA samples from mg$\Delta^{lpn}$ and dMut mice, compared to both WT control and *Hspg2${\;}^{+/-}$* mice (Figure 6I).

## 4 Discussion

Surgical intervention remains the most effective treatment option for patients suffering from an aortic aneurysm, yet resolving when best to operate poses numerous challenges (42). Appreciation of the diffuse mechanical and structural remodeling that accompanies progressive focal aortic dilatations could support clinical decision-making. However, *ex-vivo* mechanical characterization of human tissues often relies on aortic specimens excised from patients during surgery for advanced-stage aortopathy. To overcome this obstacle, animal models offer the unique opportunity to track mechanical and structural features of aortic aneurysms across all stages of the disease (43). We submit here that the mg$\Delta^{lpn}$ mouse model of Marfan syndrome (MFS) is a viable candidate for this purpose. Many snapshot studies on aneurysmal tissue mechanics leverage *Fbn1*${\;}^{mgR/mgR}$ mice (13) that exhibit a severe MFS phenotype and die spontaneously of aortic rupture by 9 weeks-of-age (44). Noting that the murine aorta reaches mechanical maturity around 8 weeks (45) deters from performing longitudinal studies in *Fbn1*${\;}^{mgR/mgR}$ mice. As an alternative, the *Fbn1*${\;}^{C1041G/+}$ mouse model of thoracic disease (13, 14) enjoys a longer life-span at the expense of a mild phenotype that does not manifest until after 9 months (46, 47). On account of the stable phenotype (16), the early presentation of thoracic aortopathy (12 weeks), and the long life-span, mg$\Delta^{lpn}$ mice therefore embody a suitable option for evaluating the progression of aneurysmal disease.

While most literature probes the mechanical response of murine aneurysmal tissues at 9 weeks due to the low survival of *Fbn1*${\;}^{mgR/mgR}$ mice beyond this age, we opted to incorporate three endpoints with the purpose of capturing pre- (4 weeks), early- (12 weeks), and advanced- (30 weeks) aortic dilatation. Since male dMut mice are not viable (1 male vs. 5 female mice born out of 170 offspring; Figure 1E), we restricted our study to females. Strong sexual dimorphism in aortic aneurysm development has been previously reported in MFS, with male mice exhibiting a worse phenotype that is more vulnerable to environmental triggers (47). Since male sex enhances the susceptibility to thoracic aortopathy (46, 48), we acknowledge that female mg$\Delta^{lpn}$ mice may experience milder aortic disease yielding lower likelihood to encounter premature death than their male litter mates (49). As such, there remains a need for elucidating sex differences in mg$\Delta^{lpn}$ mice, which we were unable to capture in this work. Regardless, the progressive shift toward a collagen-driven pressure vs. diameter response (Figure 2D) and the gradual loss of axial extensibility (Figure 2E) in the mg$\Delta^{lpn}$ ascending thoracic aorta are consistent with qualitative snapshot observations in male *Fbn1*${\;}^{mgR/mgR}$ and *Fbn1*${\;}^{C1041G/+}$ mice (12, 44, 47, 50–52). The inherent biaxial stiffening that ascending aortic tissues experience between 4 and 30 weeks-of-age (Figures 2F,G, Supplementary Material Table S1) further reflects structural findings and aligns with clinical observations (53–55). Importantly, circumferential stiffening of 4 week-old mg$\Delta^{lpn}$ tissues beyond control levels (Figure 2F) despite preserved ascending aortic caliber (Figure 2A) advocates for structural and functional remodeling to precede aneurysmal dilatation and increase the propensity thereof. Weiss et al. similarly proposed that tissue stiffening between 8 and 12 weeks-of-age may prime the *Fbn1*${\;}^{C1041G/+}$ aorta for later aneurysm formation (47).

Expected heterogeneity in the time of aortic disease onset (50) and low viability of dMut mice prompted limiting the comparison across genotypes (WT, *Hspg2${\;}^{+/-}$*, mg$\Delta^{lpn}$, and dMut) and aortic phenotypes to the 12-week endpoint. By then, 3 out of 9 mg$\Delta^{lpn}$ and 3 out of 5 dMut mice departed from the 95% confidence interval range for expected ascending aortic diameter by body mass (Figure 1G) (50, 51). To explore the effect of lumen widening on aortic function we further examined non-parametric correlations between mechanical behavior and luminal diameter. While energy storage and cyclic distensibility decline in mice carrying the mg$\Delta^{lpn}$ mutation (Figures 4H,I), these metrics only moderately or mildly correlate with ascending aortic size (Figures 5D,H). The functional impairment of the aorta as imposed by the *Fbn1* variant does not therefore reflect aneurysm progression. Note that the structural stiffening of the aorta nonetheless portends to worse clinical outcomes in MFS patients (56). Echoing our earlier inferences (12), circumferential tissue stiffness is elevated in mg$\Delta^{lpn}$ and dMut mice (Figure 4C) and most strongly correlates with ascending aortic diameter (Figure 5C), thereby alluding to severe microstructural remodeling concurrent with aneurysm formation.

Consistent with previous findings (57–62), collagen deposition and progressive loss of elastic fiber integrity contribute to medial deterioration as the ascending aorta dilates in aging mg$\Delta^{lpn}$ mice (Figure 2, Supplementary Material Figure S1). Furthermore, patterns of microstructural remodeling are comparable in all 12-week-old mice carrying the *Fbn1* variant and mostly depend on the severity of aneurysmal disease. In support of wall thickening and lumen widening, the total area of collagen compared to elastin and the relative content of cytoplasm and other non-elastin tissue elements are both larger in the aorta of mg$\Delta^{lpn}$ and dMut mice (Figures 6E,H). Noting that mechanical competence in addition to mere content supports normal aortic function (63, 64), we analyzed elastic fiber architecture in the aorta of mice carrying the mg$\Delta^{lpn}$ mutation. While normal-sized vessels exhibit a radial gradient in medial degeneration that advances from severe fiber fragmentation at the inner elastic lamina to preserved fiber integrity at the adventitial interface, the microstructural disarray encompasses the entire medial compartment in aneurysmal aortic samples (Figure 6C). Interestingly, Ferruzzi et al. noted a reverse radial gradient in elastic fiber fragmentation when characterizing aortic microstructure in the *Fbln5*${\;}^{-/-}$ mouse model of impaired elastogenesis (65). Given that *Fbln5*${\;}^{-/-}$ mice develop severe arterial elastopathy in the absence of aneurysmal dilatation or dissection (66), preserving the integrity of the elastic laminae adjacent to the lumen appears to be protective against ascending aortic dilatation. It has been suggested that an early breach of the internal elastic lamina may facilitate immune cell infiltration and perpetuate pathological remodeling that promotes aneurysm formation (67).

Large variability in phenotypic severity and intrafamilial disease manifestations across MFS patients (20, 68) rationalizes efforts to identify modifier genes and quantify their contribution to clinical outcomes. Perlecan haploinsufficiency due to heterozygous mutation in the candidate modifier gene *Hspg2* reduces the viability of mg$\Delta^{lpn}$ mice (Figures 1E,F), thereby suggesting that concurrence of the *Hspg2* and *Fbn1* variants may compromise the long term survival of patients with MFS. Interestingly, necropsies on dMut mice revealed cardiac abnormalities that were reminiscent of congestive heart failure with left atrial dilatation. This is consistent with the syndromic nature of MFS, which affects multiple organ systems and presents diverse cardiovascular complications including enlargement of the left atrium (69). Furthermore, similar to our observations in dMut mice, congestive heart failure rather than aortic enlargement is the leading cause of early mortality in neonatal MFS patients within the first 2 years of life (70). Since perlecan has been shown to play an important role in heart stability (71), concurrent *Hspg2* and *Fbn1* mutations may escalate cardiac symptoms beyond threshold for clinical relevance.

Although Nonaka et al. reported spontaneous aortic dissection in lethality-rescued *Hspg2${\;}^{-/-}$* mice by 10 weeks-of-age (28), the aorta of *Hspg2${\;}^{+/-}$* mice exhibits normal mechanical behavior at the 12-week endpoint (Figures 3 and 4). The mg$\Delta^{lpn}$ variant likewise imposes an overall stronger imprinting than perlecan haploinsufficiency on the global mechanical response of the dMut aorta at 12 weeks (Figures 3A–D). Nevertheless, the dMut but not the mg$\Delta^{lpn}$ aorta endures a decline in axial stretch (Figure 4D), which is a common early sign of maladaptive arterial wall remodeling (72). Consistent with mechanical readouts, 12 week-old dMut mice experience comparable incidence of fatal aortic dissection/rupture but nonetheless suffer from aortic dilatation at a moderately higher frequency than mg$\Delta^{lpn}$ mice. As an important note, perlecan-null mice surviving to birth display normal basement membranes that quickly deteriorate when exposed to mechanical loads, leading to premature death (73). While our *Hspg2${\;}^{+/-}$* and dMut mice express significantly lower though not negligible levels of perlecan compared to their respective controls (Supplementary Material Figure S2), it is possible that overexpression of other extracellular matrix elements may strengthen elastic fibers and protect them from fragmentation, at least early in the mouse life (74).

Mounting evidence advocates for evaluating the effect of perlecan haploinsufficiency on the mechanical response of the ascending thoracic aorta within the context of MFS (21, 28). As a first step, we performed passive inflation/extension tests to interrogate elastic and collagenous fibers as load-bearing components of the aortic wall, but neglected vasoactive responses involving vascular smooth muscle cells (vSMC). Phenotypic switching of vSMC from contractile to synthetic occurs at a higher rate in aneurysmal disease and leads to increased production of proteolytic enzymes that degrade the extracellular matrix and weaken the arterial wall (75, 76). Disruption of endothelial nitric oxide signaling is yet another common feature of thoracic aortopathies (77) that prevents vSMC relaxation and hinders flow-mediated vasodilatation (78). Perlecan notably localizes in the basement membrane of the aorta (79) where it acts as a shear flow sensor for endothelial cells (73), and perlecan deficiency reduces the expression of endothelial nitric oxide synthase (80). Further evidence suggests that perlecan and other heparan sulfate proteoglycans may influence smooth muscle cell activation and proliferation following vascular injury (81). Future efforts should therefore probe adrenergic vasoconstriction as well as endothelium-dependent and independent vasodilatation of the *Hspg2${\;}^{+/-}$* and dMut aorta, which may provide further insight on the lower viability of mice with concurrent *Fbn1* and *Hspg2* mutations. Since the effect of modifier genes often becomes apparent under stress conditions (82), it will also be important to characterize the structure and function of ascending thoracic aortic tissues from dMut mice that have been rendered hypertensive, a known risk factor for aneurysmal disease (7, 83, 84).

### 4.1 Conclusions

The mg$\Delta^{lpn}$ mouse strain carries a *Fbn1* variant that replicates in mice the classic phenotypic traits of patients with Marfan syndrome (MFS). The longer life span of mg$\Delta^{lpn}$ mice compared to alternative mouse models of MFS enabled us to examine the effect of a causative *Fbn1* mutation on ascending thoracic aorta (ATA) dilatation, tissue microstructure, and mechanical function. We then explored the effect of the candidate modifier gene *Hspg2* on the thoracic aortopathy associated with MFS by cross-breeding *Hspg2${\;}^{+/-}$* with mg$\Delta^{lpn}$ mice to create dMut mice. We show that elastic fiber fragmentation in mice carrying the mg$\Delta^{lpn}$ mutation first occurs in the proximity of the internal elastic lamina and precedes aortic dilatation. Loss of elastic fiber integrity then expands radially toward the adventitial side as the aneurysm forms and enlarges. Throughout this process, the ratio of total collagen to elastin area increases, tissues become stiffer and lose capacity for energy storage, while the ATA as a structure experiences reduced cyclic distensibility. Tissue stiffening strongly correlates with dilatation while the functional decline only moderately or mildly depends on ATA size. Perlecan haploinsufficiency reduces the overall viability of dMut mice, increases the occurrence of aortic dilatation by 12 weeks-of-age, and reduces the axial stretch, yet it does not aggravate the microstructural disarray nor the stiffening of ATA tissues at the 12-week endpoint. Further effort is therefore warranted to evaluate the effect of *Hspg2* and other modifier genes on later-stage thoracic aortopathy in MFS, with the hope of assisting the risk stratification of patients suffering from the disease.

## Data Availability

The original contributions presented in the study are included in the article/Supplementary Material, further inquiries can be directed to the corresponding author.
