# Supplementary material for: The Fbn1 gene variant governs passive ascending aortic mechanics in the mgΔlpn mouse model of Marfan syndrome when superimposed to perlecan haploinsufficiency
Source: Front Cardiovasc Med. 2024 Mar 13;11:1319164. doi: 10.3389/fcvm.2024.1319164 (PMC10965555; doi:10.3389/fcvm.2024.1319164)
Supplement: Supplementary file 1 [file Datasheet1.pdf]

Supplementary Material

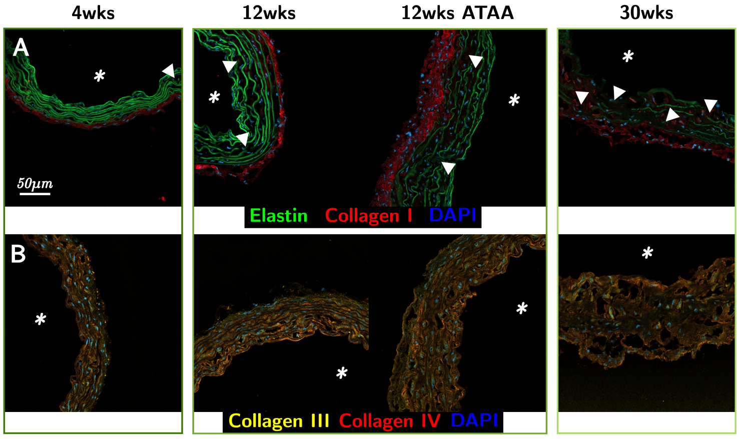

Figure S1. Elastic and collagen fiber architecture in tissues from the ascending thoracic aortic (ATA) of mgΔ^{lpn} mice at 4- (regular-sized), 12- (regular-sized and dilated), and 30- (dilated) weeks-of-age visualized by immunofluorescence staining. Tissue microstructure appears normal in ATA samples from 4-week-old mice (**A**,**B**). Elastic fiber fragmentation begins in the proximity of the internal elastic lamina in regular-sized ATA samples and extends outward toward the adventitial interface in dilated vessels at the 12-week endpoint (**A**). As the aneurysm develops by 30 weeks-of-age the entire medial architecture is in disarray, with larger gaps in the elastic fiber network and increased collagen I expression in the media compared to tissues from dilated ATA samples at 12 weeks (**A**). Collagen III and IV primarily colocalize in the aortic media in all age groups (**B**). An asterisk marks the intimal side and white triangles show fragmentation, gaps, or disarray in the elastic fiber network.

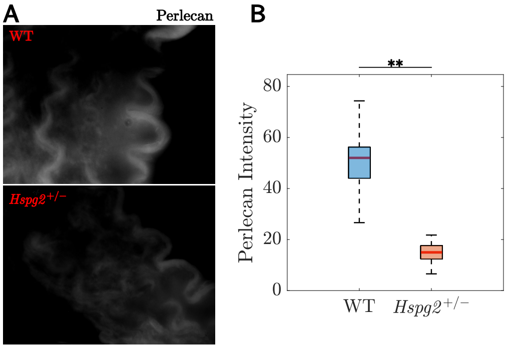

Figure S2. Immunofluorescence staining with anti-perlecan antibodies (MA1-06821 1:250) reveals a significant reduction in perlecan accumulation in both the tunica intima and the tunica media of the ascending thoracic aorta (ATA) from *Hspg2*$^{+/-}$ mice at the 12-week endpoint, compared to wild-type (WT) controls. Statistical significance denoted by overbar, with * for p<0.05 and ** for p<0.01.

Table S1. Estimated descriptors of structural and material properties for the ascending thoracic aorta (ATA) of wild-type (WT) control, *Hspg2*$^{+/-}$, mgΔ^{lpn}, and double-mutant (dMut) mice. Statistical significance across genotypes at the 12-week endpoint denoted by * relative to WT control and † relative to *Hspg2*$^{+/-}$ for $p<0.05$. Statistical significance across ages in WT and mgΔ^{lpn} mice denoted by $^\flat$ relative to the 4-week and $^\sharp$ relative to the 12-week endpoints for $p<0.05$.

	**WT**			*Hspg2*$^{+/-}$	**mgΔ^{lpn}**			**dMut**
Age (wks)	4	12	30	12	4	12	30	12
n (dilated/aneurysmal)	4	14	3	9	4	9 (3)	2 (2)	5 (3)
Distensibility (MPa^{-1})	26.18 ± 2	25 ± 1	23 ± 1	27 ± 1	12 ± 2	9 ± 1*†	7 ± 1	11 ± 1*†
Unloaded								
Outer Diameter (μm)	1025 ± 23	1151 ± 10$^\flat$	1158 ± 24$^\flat$	1134 ± 12	1103 ± 29	1381 ± 74*†	2531 ± 127$^{\flat\sharp}$	1459 ± 127*†
Wall Thickness (μm)	106 ± 6	112 ± 4	108 ± 5	101 ± 3	96 ± 2	116 ± 5	107 ± 1	120 ± 8
Axial Length (mm)	2.39 ± 0.14	2.20 ± 0.07	2.35 ± 0.13	2.28 ± 0.19	2.29 ± 0.11	3.14 ± 0.13$^{\flat,*\dagger}$	4.23 ± 0.33$^{\flat\sharp}$	2.92 ± 0.14*†
Loaded diastolic pressure								
Outer Diameter (μm)	1348 ± 4	1502 ± 21$^\flat$	1527 ± 26$^\flat$	1496 ± 23	1585 ± 45	1896 ± 114*†	3314 ± 116$^{\flat\sharp}$	2059 ± 165*†
Wall Thickness (μm)	43 ± 4	48 ± 1	44 ± 1	38 ± 2	42 ± 2	51 ± 3	49 ± 1	50 ± 4
Stretch (-)								
Circumferential	1.42 ± 0.03	1.40 ± 0.02	1.41 ± 0.06	1.41 ± 0.03	1.54 ± 0.04	1.46 ± 0.02	1.35 ± 0.02$^\flat$	1.51 ± 0.03*
Axial	1.75 ± 0.09	1.68 ± 0.02	1.75 ± 0.03	1.69 ± 0.03	1.50 ± 0.06	1.58 ± 0.04	1.63 ± 0.03	1.52 ± 0.04*†
Cauchy Stress (kPa)								
Circumferential	161 ± 23	159 ± 5	176 ± 3	180 ± 11	191 ± 7	193 ± 15	354 ± 2$^{\flat\sharp}$	199 ± 10
Axial	270 ± 33	229 ± 11	246 ± 12	318 ± 20	177 ± 30	192 ± 18	246 ± 5	222 ± 22
Linearized Stiffness (MPa)								
Circumferential	0.78 ± 0.10	0.79 ± 0.02	0.94 ± 0.04	0.85 ± 0.05	1.40 ± 0.13	1.78 ± 0.18*†	4.52 ± 0.91$^{\flat\sharp}$	1.56 ± 0.16*†
Axial	1.36 ± 0.11	1.19 ± 0.09	1.22 ± 0.06	1.19 ± 0.10	0.94 ± 0.17	1.18 ± 0.11	1.78 ± 0.31$^\flat$	1.01 0.10
Stored Energy (kPa)	66 ± 12	55 ± 3	59 ± 2	61 ± 5	42 ± 5	41 ± 3*†	48 ± 8	40 ± 5†
Loaded systolic pressure								
Outer Diameter (μm)	1514 ± 55	1678 ± 23$^\flat$	1691 ± 48$^\flat$	1688 ± 27	1674 ± 34	1979 ± 115*†	3425 ± 101$^{\flat\sharp}$	2168 ± 163*†
Wall Thickness (μm)	38 ± 3	42 ± 1	39 ± 1	38 ± 2	40 ± 2	49 ± 3†	47 ± 2	50 ± 4†
Stretch (-)								
Circumferential	1.61 ± 0.02	1.58 ± 0.02	1.58 ± 0.06	1.60 ± 0.03	1.63 ± 0.03	1.53 ± 0.02	1.40 ± 0.03$^{\flat\sharp}$	1.59 ± 0.04
Axial	1.75 ± 0.09	1.68 ± 0.02	1.75 ± 0.03	1.69 ± 0.03	1.50 ± 0.06	1.58 ± 0.04	1.63 ± 0.03	1.52 ± 0.04*†
Cauchy Stress (kPa)								
Circumferential	312 ± 47	304 ± 10	330 ± 8	350 ± 22	322 ± 15	318 ± 25	568 ± 4$^{\flat\sharp}$	334 ± 19
Axial	338 ± 46	296 ± 14	311 ± 9	318 ± 20	219 ± 36	236 ± 22†	337 ± 10	225 ± 22†
Linearized Stiffness (MPa)								
Circumferential	1.89 ± 0.27	1.87 ± 0.09	2.22 ± 0.08	2.18 ± 0.19	4.51 ± 0.67	4.83 ± 0.57*†	10.18 ± 0.25$^{\flat\sharp}$	4.51 ± 0.50*†
Axial	1.75 ± 0.19	1.58 ± 0.14	1.57 ± 0.09	1.58 ± 0.13	1.18 ± 0.19	1.51 ± 0.15	2.63 ± 0.45$^{\flat\sharp}$	1.32 ± 0.13
Stored Energy (kPa)	94 ± 17	81 ± 3	85 ± 2	93 ± 7	56 ± 8	52 ± 5*†	63 ± 10	54 ± 7*†
